# Polymorphism Data Can Reveal the Origin of Species Abundance Statistics

**DOI:** 10.1371/journal.pcbi.1000359

**Published:** 2009-04-24

**Authors:** Yosef E. Maruvka, Nadav M. Shnerb

**Affiliations:** Physics Department, Bar-Ilan University, Ramat-Gan, Israel; University of Washington, United States of America

## Abstract

What is the underlying mechanism behind the fat-tailed statistics observed for species abundance distributions? The two main hypotheses in the field are the adaptive (niche) theories, where species abundance reflects its fitness, and the neutral theory that assumes demographic stochasticity as the main factor determining community structure. Both explanations suggest quite similar species-abundance distributions, but very different histories: niche scenarios assume that a species population in the past was similar to the observed one, while neutral scenarios are characterized by strongly fluctuating populations. Since the genetic variations within a population depend on its abundance in the past, we present here a way to discriminate between the theories using the genetic diversity of noncoding DNA. A statistical test, based on the Fu-Li method, has been developed and enables such a differentiation. We have analyzed the results gathered from individual-based simulation of both types of histories and obtained clear distinction between the Fu-Li statistics of the neutral scenario and that of the niche scenario. Our results suggest that data for 10–50 species, with approximately 30 sequenced individuals for each species, may allow one to distinguish between these two theories.

## Author Summary

One purchases 100 wineglasses and 100 pairs of pants. After one year, 10 glasses and 10 pants survive. What can be said about the relative quality of the survivors? Well, clothes “die” as a result of accumulated wear; the surviving items are of better quality. The breaking of a wineglass is an external, random event: here the survivors are not the best, but the luckiest. To tell apart the superior from the fortunate, one should examine the development over time: the number of surviving items decays exponentially with time for the glasses and follows a sigmoid curve for the pants. An ongoing argument among macroecologists deals with similar issues. Adaptive theories suggest that the frequent species are the fittest, while the neutral theory explains the observed frequencies as a result of demographic stochasticity, assuming all species to have the same fitness. The histories suggested by the two scenarios are clearly different, but how can one probe the prehistoric abundance of species? In fact, past abundance is reflected in current genetic variance within a population. Here, we present a new technique, based on the Fu-Li F-statistic, which allows one to distinguish between niche and neutral scenarios and to resolve this important debate.

## Introduction

One of the most interesting peculiarities of mother nature is the large variance in abundance of otherwise similar species. In the tropical rainforest, for example, there are differences of 4–5 orders of magnitude in the observed abundance of tropical trees [1]–[5]. Moreover, the abundance distribution admits a fat tail, which may be described by power-law or log-normal statistics. This observation is somewhat puzzling, as on the basis of evolutionary mechanisms and the competitive exclusion principle one expects the survival of only a few, most fit, species.

The simplest explanations for this phenomenon are based on “niche theory” [5]–[7]. This theory suggests that the abundance differences reflect fitness, or competitive ability variations. Strong species defeat the weak, and thus their population is large; weaker species survive due to geographical variations (regions where their fitness is better), symbiosis with strong species, or spatio-temporal fluctuations of the environmental conditions. Mathematically speaking, the system may be described by a series of coupled differential equations of, say, the Lotka-Volterra type, where each of the species undergoes logistic growth, but the growth rate and the carrying capacity are determined by the abundance of other species. The actual abundance distribution reflects a stable fixed point of this set of equations. In a fixed environment, thus, the abundance ratios among species are fixed up to demographic stochasticity; if the deterministic equations predict population size 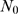 for certain species, one should expect temporal fluctuations proportional to 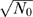. The observed frequency at present reflects the intrinsic fitness of that species, and thus one conjectures a similar community structure in past generations.

Another theory that gained much popularity in the last decade is the neutral theory of species abundance. It assumes [1],[2] that the fitness differences between species are negligible, and that the system is controlled solely by demographic stochasticity. The underlying dynamic that controls the abundance of different trees in the tropical forest is similar to the dynamic that governs surname frequency. The fact that there are many “Smith”s but only a few “Maruvka”s does not reflect (we hope) the undesirable features of the infrequent surname, but rather the stochastic inheritance, appearance (mutation) and “death” of surnames along genealogic lineages [8].

**Figure 1:**
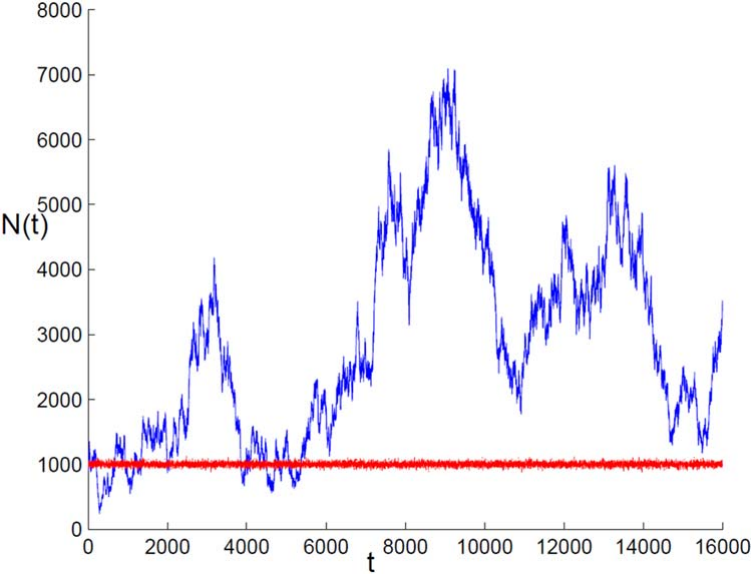
Abundance Histories. Typical abundance histories for the neutral theory (blue) and for the niche theory (red), as obtained from the simulation procedure described in the text. For the two cases, the current population is 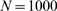, but the histories suggested by the two models are completely different: niche history is characterized by bounded fluctuations around a fixed value, while neutral history fluctuates strongly and admits periods of high abundance, bottlenecks and so on. Clearly, the “niche” history shown here is an idealization, as it assumes fixed environmental conditions; in reality one should expect larger, environmentally driven, abundance fluctuations. The possible effects of environmental stochasticity are discussed below.

Within the framework of the neutral model, demographic stochasticity may be described as a multiplicative random walk along the abundance line. Multiplicative random processes are known for many years as the underlying mechanism behind fat-tailed statistics, e.g., firm size distributions [9],[10]. In fact, niche models for species abundance, like MacArthur's broken stick [6] or May's independent factor explanation [7] , are also based on some sort of multiplicative process. The difference we intend to extract here is that in the neutral scenario this random process characterizes the actual *time evolution* of species abundance, while the adaptive theories assume such a process in the fitness/resource space. Thus, if niche-based theories are correct, the real-time stochastic birth-death process is biased towards the observed (present) frequency 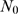. If the neutral theory is right, the random walk is almost unbiased (a tiny bias towards extinction is related to the mutation rate), and the species frequency undergoes huge fluctuations. An illustration of the temporal abundance fluctuations for the two scenarios is given in Figure 1.

Confronting the different models on the basis of current community structure data poses a very difficult statistical problem [5], [11]–[13]. Even in the presence of a reliable datasource, distinguishing between the various fat-tailed distributions (e.g., zero-sum multinomial, multivariate Poisson lognormal, broken stick distribution, etc.) is a demanding task. The noisy measurement of relative abundance in ecosystems renders this analysis even harder to accomplish. On the other hand, it would be very easy to recognize the underlying mechanism if the history of the frequency variations was given, as seen in Figure 1. Unfortunately, the Neanderthal men were too busy to conduct large-scale surveys of species abundance. In order to gather the relevant information one must seek out traces of the past in the present, i.e., the genetic polymorphism of the community.

In this work we present an experimental method that extracts these differences and allows one to distinguish between the two scenarios. It requires the collection of a large amount of genetic data from the current population, in particular noncoding DNA from either haploid (mtDNA, Y-chromosome or cpDNA) or diploid sequences. Intuitively, the genetic diversity of these sequences should reflect the history of the species abundance; one expects different results for a more or less fixed population (as suggested by the niche theory), than for a strongly fluctuating population with bottlenecks and high prevalence times (as suggested by the neutral theory). Here we quantify this concept, explain how to distinguish between the two scenarios, and demonstrate our results in a numerical experiment using “DNA sequences” obtained from simulated data with different histories.

Our technique is limited by two time scales. The sequence mutation time sets its resolution, as no reliable conclusion may be drawn on the basis of only a few mutations. The abundance history may be recovered for timescales that are much larger than the typical time needed for a single mutation to appear in the whole sequence. The time to the most recent common ancestor sets, of course, the maximal timescale. For an almost fixed population (niche scenario) of size 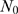, the most recent common ancestor of any typical collection of sampled individuals appears about 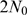 generations before present. This implies that our method, which uses the “structure” of the phylogenetic tree, enables differentiation between scenarios if the abundance differences were substantial in the last 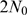 generations. Accordingly, our techniques are not limited to small, local ecosystems but are applicable to the metacommunity as well, since the “time horizon” to the past is proportional to the abundance.

A similar idea, utilizing the differences in assumed history to distinguish between the two hypotheses, was suggested by Ricklefs [14] (see also similar approach used in [15]). Relying on data from passerine birds, Ricklefs compared the species' lifetime (i.e., the time elapsed since the species first appeared) and its contemporary abundance. Under the assumption of neutrality, the average species' lifetime is almost linearly proportional to the current abundance (technically, this is the first passage time [16] of a multiplicative random walk started at 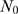). According to Ricklefs, [14] the species' actual lifetime (obtained from genetic divergence data) is much shorter than expected by Hubbell's neutral theory. His method, however, requires prior knowledge of the current population size and mutation rate, two parameters that may be difficult to obtain. Here we suggest a method that only requires knowledge of the genetic variability.

Before we discuss the polymorphism analysis itself, let us add an important comment. Restricting our considerations to “pure” adaptive/neutral histories, like those demonstrated in Figure 1, is clearly a simplification. In reality , one should expect, for example, larger fluctuations for an ecosystem that obeys the rules of the niche theory, due to the effects of *environmental* stochasticity. We do assume, however, that these fluctuations either conserve the species abundance ratio (i.e., are not species specific) or are relatively weak. If environmental fluctuations cause rapid shifts in the relative species frequency, the conceptual meaning of the “niche theory” becomes unclear and the difference between the two scenarios is not so interesting. Throughout this work we therefore assume that the effect of environmental stochasticity is weak and yields only minor corrections to the niche/neutral predictions. In the final section we return to this issue, and discuss in detail the various types of environmental stochasticity, together with their identification using genetic polymorphism data.

## Results/Discussion

### Fu-Li Statistic

We tried a number of methods in order to distinguish between the genetic polymorphism of the two scenarios, and found that the most efficient one is Fu & Li F-statistic [17]. Originally, this method was developed in order to measure the similarity of a given phylogenetic tree to the one expected from the Kingman Coalescent Model [18],[19]. It was used by Sjödin [20] to measure when fluctuations in the population size cancel the similarity with the Kingman Coalescent. Here we used this method in order to distinguish between the two scenarios of fluctuating populations.

The Fu-Li F-statistic compares the sum of the lengths of the external branches to the *average* internal branch length. Under the correct scaling, these lengths should be the same, if the assumptions of the Kingman Coalescent Model (fixed population size, small sample size, and neutrality of mutations) are fulfilled. Therefore, in the Wright-Fisher process, for example, the value of the F-Statistic is zero. In a growing population, this value is negative, and for a shrinking population it is positive.

Basically, the Fu-Li F-statistic compares the features of the *recent past*, which affect the external branch length, to the features of the *far past*, affecting the internal branches. Thus, it emerges as a suitable technique for distinguishing between the two scenarios. In the niche scenario, the population in the past is similar to the population in the present, so the statistic should be approximately zero. For a neutral scenario, the population in the present differs from the past population; in most cases, the population in the present is larger than the population in the past (this is an interesting feature of a multiplicative random walk with an absorbing state, see [21]). Therefore, one expects that the statistic for that scenario will admit a broad distribution with a negative average.

The F-statistic is defined by:
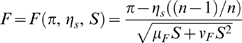
(1)where 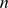 is the sample size, 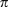 is the average number of pairwise nucleotide differences (the average being over all possible pairs in the sample), S is the number of segregating sites, 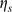 is the number of singletons (mutations that appear in only one individual in the sample), and 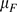 and 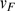 are constants given the sample size 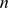.

We also worked out the Fu and Li D-statistic for the same datasets. The results were similar to those of the F-statistic but the resolution obtained from the F-statistic was better and is therefore preferable.

### Differentiating between the Two Scenarios

**Figure 2:**
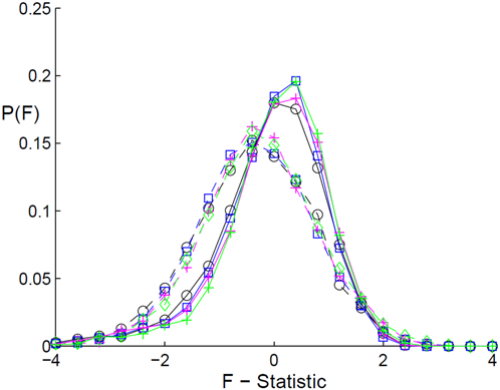
Fu-Li *F-Statistics*. The distributions of the Fu-Li *F-Statistics* for the two different scenarios are presented. The full lines correspond to histories that obey the rules of the niche history, while the dashed lines represent the statistics gathered from neutral histories. Several current population sizes are presented. Current population size 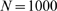 is represented by black circles, 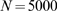 by blue squares, 10000 by magenta+signs, and 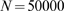 by green diamonds. It can be seen that there is almost no difference between current population sizes with the same history; the entire discrepancy is between the two scenarios. Every distribution was produced by 3000–5000 realizations, and from every realization 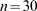 individuals were sampled. In fact, only when we decreased the sample size to 10 individuals per species did the statistical measure really fail.

We performed many numerical experiments simulating the niche scenario and the neutral scenario, and calculated the F-Statistic for each realization. We then produced the probability distribution of the F-Statistic for the two types of histories. As can be seen in Figure 2 the F-statistic differs in the two scenarios; both the width of the distribution and its average are not the same, as expected. An important feature of these statistics is that they do not depend on the species' abundance, only on their history.

Given real DNA sequences from several species, this difference in the distributions can be used to determine whether the species followed the *Niche* history or the *Neutral* history, and end the ongoing debate between these two hypotheses.

Since we do not currently have enough DNA sequences from many different species, we did not try to check the common method of 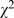 test, that given a few data points can distinguish between two similar distributions, rather we give here only a rough estimation for the number of species needed to distinguish between the two hypotheses. For 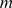 species, the standard error of the F-statistics is 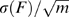, and in order to discriminate between the two scenarios this quantity should satisfy:
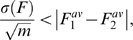
(2)where 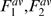 are the averages of the F-statistic of the neutral and niche scenarios respectively. For a sample size of 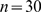 individuals per species, as in Figure 2, the required number of species necessary to decide between the two theories is 10–50.

While our analysis until this point assumed one independent community, i.e. meta-community [2], our approach can also be applicable to local communities. For local communities (like those described by an island-mainland model), in cases of weak migration, the abundance fluctuations for neutral population are still much larger than those expected from the niche theory and one can still distinguish between the two scenarios. The migration is “weak” when the relaxation time towards the metacommunity's relative abundance is large relative to the time scale associated with the demographic stochasticity - this is the limit 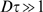 considered by [22]. Moreover, if there is a possibility to recover the migration rates from the abundance data (e.g., in the case of a few local communities coupled to the same metacommunity, such that the parameter 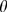 used in [1],[2] is the same for all islands), one can calculate the Fu-Li statistic for different local communities with different migration rates. This statistic should approach the neutral case as the migration rate gets smaller.

### Additional Polymorphism-Based Techniques

In this section we present a short survey of other methods we examined to distinguish between niche and neutral histories. At the end of the day we concluded that the Fu-Li method is superior if no information is given beyond the genetic data. Yet, in the presence of other pieces of information, one of the following techniques may be preferable to the Fu-Li method.

#### Time to most recent common ancestor (TMRCA)

The time to most recent common ancestor admits different statistical properties in the two scenarios. For 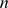 individuals out of a *fixed* population of size N, the Wright-Fisher theory predicts that the TMRCA is 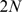, and for the niche scenario the result is more or less the same. In contrast, for neutral histories the TMRCA is affected by “bottlenecks” (times in the past for which the population was small) and on average is much smaller than in the niche scenarios. TMRCA can therefore be used to distinguish between the two hypotheses.

The difficulty with this method is that the TMRCA scales with the population size, and so this number must be known for any species. Moreover, what should be known is not the total population of a species, but the “effective population” of individuals (i.e., the number of animals/trees that may be the parents of an individual offspring). We must add that if this effective population size is somehow known (e.g., for a small, isolated island where the population is well mixed) this method may be superior because the sample size needed from each species is small (10 or less).

#### Number of lineages as a function of time

The average number of lineages as a function of time, 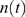, (back in the past starting with sample size 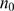 sequenced in the present) can be calculated easily for a fixed population. This number, for a set of sampled sequences, can be derived from any tree building algorithm (the dependence of 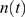 on the specific algorithm used is very weak). Again, the niche scenario is similar to a fixed population history, while the neutral scenario is different. Fitting 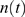 with the analytical results (obtained for fixed population) is thus much better for the niche scenario.

The weak point of this method lies in the fact that the goodness of fit changes with the population size and again requires an a-priori knowledge of the population's effective size.

#### Family size distribution

An interesting idea is to use the abundance statistics of “genetic families” in order to learn about the species abundance. We defined a “family” as all the individuals with exactly the same genome (any mutation is an establishment of a new family). If the population is fixed, the family size distribution should obey the same abundance statistics of the neutral theory, as this is simply a neutral process with mutations.

It turns out, however, that there is almost no difference between the niche and the neutral histories in the family size distribution. The reason for this is that the family size distribution depends only on the close history (recent times), and in this period the two scenarios admit the same population size. We hope to discuss this issue in a separate work.

### Environmental Stochasticity

As we have mentioned above, the “pure” niche/neutral scenarios considered here are an idealization that may be true in some cases (e.g. small communities), but in other cases one should expect large fluctuations in the abundance of a species due to environmental stochasticity. Indeed, for certain ecosystems (like phytoplankton) some degree of environmental fluctuations has been suggested in order to explain, in the framework of niche theory, how the system overcomes the competitive exclusion principle [23]. It is thus important to consider the conceptual and the practical implications of this stochasticity in both frameworks. We stress that the following discussion is relevant not only to the polymorphism-based technique presented here, but also to the niche-neutral debate in general, including the analysis of the observed species abundance ratios.

One fundamental observation is that all theories become practically neutral in the limit of *large*, *fast and independent* environmental fluctuations. If the fitness of a species varies tremendously in time, and is uncorrelated with the (also fluctuating) fitness of other species, and if the correlation time of these fluctuations approaches zero, the fitness differences are averaged out and the adaptive dynamics is equivalent to the neutral one. Niche theories are meaningful only if (at least) one of the three conditions mentioned above is not satisfied: the environmentally-induced fitness fluctuations should be either weak, slow, or correlated.

If the effect of environmental stochasticity is weak, the corrections to the predictions of the “pure” theory are relatively small. In that case one may use our Fu-Li technique, expecting only small deviations from the predictions for the idealized case. In other occasions, however, one can find evidence for large perturbations (e.g., climate changes) that affect the ecosystem. Here the timescale is important: one may try to relate observed quantities (abundance ratios, genetic polymorphism) to the predictions of an adaptive theory only if the characteristic time needed for the ecosystem to reach demographic equilibrium is much shorter than the typical period between environmental shifts. The Fu-Li statistic in that case will fit our predictions if the genetic time horizon, 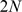, is smaller than the characteristic period between environmental transitions.

Even if these conditions are not satisfied and the Fu-Li statistic (as well as the species abundance ratio) fails to follow the pure scenario predictions one may still uses other polymorphism based methods. The basic challenge, now, is to discriminate between the neutral scenario suggested by [24], with a neutral drift superimposed on the overall carrying capacity fluctuations, and between *two* competing niche scenarios. One can imagine an adaptive ecosystem that is subject to *uniform* (correlated) pressure, such that the abundance of all species shrinks or grows in the same proportions (the relative abundance is conserved and is independent of the total population size). On the other hand, the pressure may be uncorrelated (niche-selective), not affecting all species in the same manner, in which case the abundance ratio is time-dependent [23],[25].

As suggested above, polymorphism data may be used in order to calculate 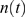, the number of lineages as a function of time, and from this quantity 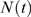 can be calculated [note that the rate of disappearance of lineages in the Wright-Fisher coalescence model is proportional to the abundance 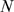]. This technique may be used in order to extract past abundance ratios for different species. With the abundance history at hand one can distinguish between the three different possibilities. In the case of niche scenarios with uniform pressure, one expects the abundance-ratio to be fixed in time. The neutral scenario suggests that the abundance ratio is not varying but 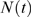 for different species is correlated, i.e., a global catastrophe resulted in a decrease of all the species and vice versa. An uncorrelated historic abundance (i.e., both abundance of any species and the abundance ratio fluctuate in time) corresponds to niche-selective pressure. Thus, even in the case of large environmental stochasticity, more sophisticated genomic techniques can be used to differentiate between the two histories.

### Summary

Most of the empirical tests suggested for the niche-neutral debate rely on snapshots, such as via comparison of the predicted and the observed species abundance ratio. Some authors did consider historic abundance data [25]–[27], but the populations they dealt with are relatively small. Moreover, these authors tested only the neutral hypothesis against the data; in order to have a well defined niche theory, one must clarify the relative weight given to the stochasticity in comparison with the deterministic part of the dynamics. In this work, we suggest the use of current genetic polymorphism as an indicator for past abundance fluctuations. We believe that due to the fast-paced development of sequencing techniques, this data will be available for analysis in the near future. By sequencing more and more individuals from different species, one may use our technique to improve the quality of the results in any ecosystem and for a large time horizon. As explained in the last section, the results may be used as a test for both niche and neutral scenarios, and may allow one to establish a “mixed” theory, comparing the importance of stochasticity vs. deterministic dynamics.

## Methods

### Simulation Technique

We have gathered our data from a simulation of the Wright-Fisher model with discrete generations. We initiate the system with 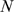 individuals, each carrying a “genome” of 1000–10000 sites. At each generation, any of the individuals produces 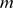 offspring, where 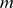 is a random number generated from a Poissonian distribution with an average of 2. Each of the offspring carries the exact DNA sequence of its ancestor with probability 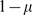, and mutates at a single, randomly chosen site with probability 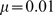. From all of the offspring in a generation, only 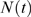 are selected at random to survive, where 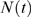 is the (time dependent) carrying capacity. For niche histories, 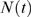 fluctuate around 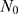 with 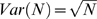, as expected for a population with a well-defined carrying capacity subject to demographic stochasticity. For neutral histories, the population size 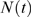 follows a Markovian process: given 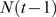, the carrying capacity of the last generation, 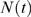 is chosen at random from a Poissonian distribution with average 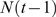. In order to compare the predictions of the two theories for a species given the current abundance 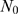, we have created first the 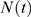 sequence starting from 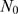 and go backwards in time up to 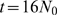. We then simulate the genealogic process from past to present, and obtain the Fu-Li statistics using the algorithm presented in [28]. For 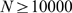 a different simulation technique has been used: the genealogic tree has been generated from the sampled population at present using the “ball in a box” procedure [29], an implementation of the Wright-Fisher process. The number of “boxes” changes from generation to generation according to the above mentioned procedure for the corresponding scenario. As seen in Figure 2 below, the Fu-Li statistic obtained using the two procedures are essentially the same.
